# Quality analysis of prior systematic reviews of carpal tunnel syndrome: an overview of the literature

**DOI:** 10.1590/1516-3180.2021.1020.R2.10102022

**Published:** 2022-12-19

**Authors:** Marcelo Cortês Cavalcante, Vinicius Ynoe de Moraes, Guilherme Ladeira Osés, Luis Renato Nakachima, João Carlos Belloti

**Affiliations:** I MD. Physician, Department of Orthopedics and Traumatology, Discipline of Hand and Upper Limb Surgery, Escola Paulista de Medicina (EPM), Universidade Federal de São Paulo (UNIFESP), São Paulo (SP), Brazil.; II MD, PhD. Professor, Department of Orthopedics and Traumatology, Discipline of Hand and Upper Limb Surgery, Escola Paulista de Medicina (EPM), Universidade Federal de São Paulo (UNIFESP), São Paulo (SP), Brazil.; III MD. Physician, Department of Orthopedics and Traumatology, Discipline of Hand and Upper Limb Surgery, Escola Paulista de Medicina (EPM), Universidade Federal de São Paulo (UNIFESP), São Paulo (SP), Brazil.; IV MD, PhD. Professor, Department of Orthopedics and Traumatology, Discipline of Hand and Upper Limb Surgery, Escola Paulista de Medicina (EPM), Universidade Federal de São Paulo (UNIFESP), São Paulo (SP), Brazil.; V MD, MSc, PhD. Adjunct Professor, Department of Orthopedics and Traumatology, Discipline of Hand and Upper Limb Surgery, Escola Paulista de Medicina (EPM), Universidade Federal de São Paulo (UNIFESP), São Paulo (SP), Brazil.

**Keywords:** Evidence-based medicine, Quality control, Carpal tunnel syndrome, Quality, Systematics reviews, PRISMA

## Abstract

**BACKGROUND:**

Carpal tunnel syndrome (CTS) is a common condition greatly affects patients’ quality of life and ability to work. Systematic reviews provide useful information for treatment and health decisions.

**OBJECTIVE:**

This study aimed to assess the methodological quality of previously published systematic reviews on the treatment of CTS.

**DESIGN AND SETTING:**

Overview of systematic reviews conducted at the Brazilian public higher education institution, São Paulo, Brazil

**METHODS:**

We searched the MEDLINE and Cochrane Library database for systematic reviews investigating the treatment of CTS in adults. The Preferred Reporting Items for Systematic Reviews and Meta-Analyses (PRISMA) and measurement tool to assess systematic reviews **(**AMSTAR) were applied by two independent examiners.

**RESULTS:**

Fifty-five studies were included. Considering the stratification within the AMSTAR measurement tool, we found that more than 76% of the analyzed studies were “low” or “very low”. PRISMA scores were higher when meta-analysis was present (15.61 versus 10.40; P = 0.008), while AMSTAR scores were higher when studies performed meta-analysis (8.43 versus 5.59; P = 0.009) or when they included randomized controlled trials (7.95 versus 6.06; P = 0.043). The intra-observer correlation demonstrated perfect agreement (> 0.8), a Spearman’s correlation coefficient of 0.829, and an ICC of0.857. The inter-observer correlation indicated that AMSTAR was more reliable than PRISMA.

**CONCLUSION:**

Overall, systematic reviews of the treatment of CTS are of poor quality. Reviews with better-quality conducted meta-analysis and included randomized controlled trials. AMSTAR is a better tool than PRISMA because it has a better performance and should be recommended in future studies.

**REGISTRATION NUMBER IN PROSPERO:**

CRD42020172328 (https://www.crd.york.ac.uk/PROSPERO/display_record.php?ID=CRD42020172328)

## INTRODUCTION

Median nerve compression in carpal tunnel syndrome (CTS) affects 1–3 people per 1,000 according to studies in the United States. This syndrome leads to pain, decreased sensitivity, and hand strength, and has a significant detrimental economic impact.^1^ The initial treatment of the condition is usually non-operative, and surgical treatment is reserved for cases in which non-surgical treatment fails or when facing advanced disease.^2^

In this context, the aims of CTS treatment include the achievement of more efficient resolution of symptoms and earlier return to work. In recent decades, many studies have been conducted to establish the best treatment for this disease. The advent of systematic reviews and modern methods of statistical evaluation is currently pushing research towards more reliable evidence. However, systematic reviews do not always follow the necessary methodological concepts, leading to imprecision and erroneous conclusions.^3^ Recent studies have shown, both in hand surgery as a whole,^4^ and specifically in carpal tunnel syndrome treatment,^5^ that systematic reviews are often lacking in quality.

To identify poorly conducted systematic reviews, objective tools and questionnaires have been developed to improve the methodological robustness of reviews and to provide a parameter for data collection, analysis, and synthesis of the evidence achieved. These protocols^6-9^ act as safeguards for systematic reviews, and numerous studies in the literature have supported their systematic usefulness.

## OBJECTIVE

This study aimed to assess the methodological quality of previously published systematic reviews on the treatment of CTS, as well as to verify the reproducibility of the A Measurement Tool to Assess Systematic Reviews (AMSTAR) and Preferred Reporting Items for Systematic Reviews and Meta-Analyses (PRISMA) scores in this scenario, as no study in the literature has previously used these two tools for this purpose.

## METHODS

The methodology of this review is registered in the PROSPERO database CRD42020172328 (https://www.crd.york.ac.uk/PROSPERO/display_record.php?ID=CRD42020172328).

### Literary search

A comprehensive literature search was performed in the MEDLINE and Cochrane Library databases for articles published from January 1950 to February 2020, with the only restriction being articles in the Mandarin language. The search strategy was performed using two methods.

Method 1 – Search for the terms “carpal tunnel syndrome” and “systematic review” in the “Clinical Queries” section of the PubMed platform. (“carpal tunnel syndrome” AND “systematic review”) AND (Therapy/Broad[filter])

Method 2 – Search with the keyword “carpal tunnel syndrome” and “systematic review” in the Cochrane Library platform with the filter “Other reviews” (Epistemonikos)

(“carpal tunnel syndrome” AND “systematic review”) AND (Epistemonikos[filter])

The results of both search strategies were independently analyzed by two researchers (M.C.C. and G.L.O.), and any discrepancies and disagreements were resolved with the help of a senior third author (V.Y.M.). We selected the MEDLINE and the Cochrane Library databases for their worldwide audience and to include relevant research data.

### Inclusion criteria

Systematic reviews (with or without meta-analysis) that included any studies (Randomized Clinical Trials or non-Randomized Clinical Trials) evaluating the treatment of CTS in an adult population (18 years or older).

### Exclusion criteria

Reviews lacking a transparent literature search and strategy for their data approach, those that were diagnostic-focused, involved anesthetic procedures, or were clearly narrative.

### Methodology evaluation (internal validity) and quality reports

The data from all evaluated studies were considered for the elaboration of a descriptive table presenting the various characteristics of the systematic reviews on the topic.

The following were included in the data analysis: journal impact factor (high impact versus low impact), performed a meta-analysis or not, number of institutions involved, total number of patients, total number of words, presence of conflicts of interest, country of origin of the study, citation of PRISMA, and inclusion or exclusion of randomized controlled trials.

### Impact factor stratification

The impact factor is expressed as the average number of weighted citations received in the last three years of articles published in the journal. This calculation yields a number, and all grades are ranked in quartiles according to the criteria of the SCImago Journal and Country Rank (https://www.scimagojr.com/journalrank.php). The evaluated journals were dichotomized between those in the first quartile (Q1), defined as high-impact publications, and those outside of this quartile (not Q1), which were defined as low-impact.

### Tools to assess quality

AMSTAR^8^ was used to assess the quality of the systematic reviews. This tool covers 16 dichotomous questions relevant to the internal validity of systematic reviews related to study design (Q1), research and study inclusion/exclusion (Q2-5), study characteristics (Q6), internal validity of systematic reviews (Q7-15), and conflicts of interest (Q16). AMSTAR has a maximum score of 16 points, with higher scores indicating better quality. This tool further grades the quality of the analyzed studies as “very low”, “low”, “medium”, or “high”.

PRISMA7 (https://www.prisma-statement.org/PRISMAStatement/) is a tool comprising 27 items that aids in the formulation and analysis of systematic reviews and meta-analyses. For this analysis, we considered all 27 items and the sum of answers as the final score. Although the overall aim of PRISMA is to help ensure the transparency of systematic reviews, in this study, it was used as a tool in which the sum of its items denoted better quality in the studies, as has been performed in previous studies.^10,11^

The acquisition of study data and application of the AMSTAR and PRISMA questionnaires were performed in duplicate. A senior author (V.Y.M.) mediated any cases of disagreement between the examiners.

### Data analysis

We defined *a priori* subgroups for a comparative analysis of the quality of systematic reviews: high-impact journal (Q1) versus low impact (non-Q1), presence of meta-analysis versus non-meta-analysis, randomized controlled trials versus non-randomized clinical trials, statement of interest versus non-declaration, whether PRISMA was cited, country of origin, and number of words.

We defined *a priori* subgroups for a comparative analysis of the quality of systematic reviews, as follows: high-impact journals (Q1) versus low-impact journals (non-Q1), presence of meta-analysis versus its absence, systematic reviews of randomized clinical trials versus studies that did not employ them, presence of a declaration of interest versus its absence, whether PRISMA was mentioned, country of origin of the study, total number of words, total number of patients, and number of institutions involved.

### Statistical analysis

Continuous variables were compared using the Mann-Whitney U test. Categorical variables were compared using the Wilcoxon’s test. Intraobserver agreement was assessed using Spearman’s correlation coefficient and the intraclass correlation coefficient. Inter-observer agreement was performed according to the Blant Altman and Kappa coefficient, with a score of more than 0.8 indicating perfect agreement; 0.61–0.8, substantial agreement; 0.60–0.41, moderate agreement; and scores below 0.4 indicating low agreement.^12^

## RESULTS

In this systematic review, we considered 55 studies.

**Figure 1.**
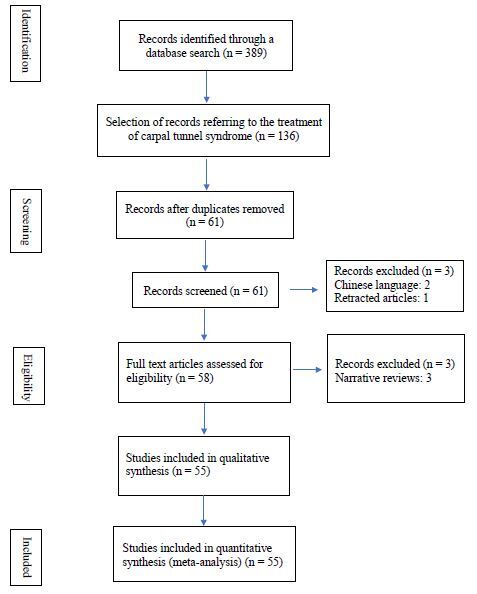
The Preferred Reporting Items for Systematic Reviews and Meta-Analyzes (PRISMA) flowchart of this study.

The PRISMA flowchart, including the reasons for exclusion at each stage, is outlined in Figure 1. Studies characteristics are detailed in Table 1,13-66 and quantitative data are presented in Table 2.

**Table 1.** Study characteristics

Author, Year	Impact factor	Conflict of interest	Country of origin	What comparison?	Number of institutions	Total number of patients	Number of words	Study design	Meta-analysis	Quoted PRISMA?
Alvayay et al.,^13^ 2008	Q4	NO	CHILE	Different CTS physiotherapeutic treatments	1	1,415	1,702	RCT, SYSTEMATIC REVIEW	NO	NO
Babaei-Ghazani et al.,^14^ 2017	Q1	NO	IRAN	Corticosteroid injection into the carpal tunnel: guided ultrasound versus guided landmark	3	137	3,502	RCT	YES	NO
Ballestero-perez et al.,^15^ 2016	Q1	YES	SPAIN	Effectiveness of nerve glide exercises in CTS	5	733	2,420	RCT, CT	NO	YES
Bekhet et al.,^16^ 2017	Q2	YES	EGYPT	Low frequency laser versus placebo	6	473	2,731	RCT	YES	YES
Burger et al.,^17^ 2017	Q2	YES	SOUTH AFRICA	Low frequency laser versus placebo	5	614	4,897	RCT	NO	NO
Burton et al.,^18^ 2016	Q1	YES	UNITED KINGDOM	Clinical course and prognostic factors of conservative treatment CTS	1	2.639	4,490	COHORT	NO	NO
Chapell et al.,^19^ 2003	Q1	YES	UNITED STATES	Neurolysis and epineurotomy versus placebo in surgical treatment	1	390	2,925	RCT	YES	NO
Chen et al.,^20^ 2014	Q1	NO	CHINA	Open versus endoscopic release	1	1.395	2,481	RCT	YES	NO
Chen et al.,^21^ 2015	Q2	YES	TAIWAN	Different local infiltrations of corticosteroids in the carpal tunnel	4	633	4,728	RCT	YES	NO
Choi et al.,^22^ 2018	Q1	YES	SOUTH KOREA/UNITED STATES	Acupuncture and related interventions for placebo treatments	2	869	13,354	RCT/QUASI-RCT	YES	YES
Dunn et al.,^23^ 2017	Q2	YES	UNITES STATES	Labor compensation versus no labor compensation in the treatment of CTS	2	4,367	2,006	PROSPECTIVE, RETROSPECTIVE	NO	NO
Franke et al.,^24^ 2017	Q1	YES	NETHERLANDS	Benefits of low frequency laser therapy for CTS	2	1,048	4,923	REVIEWS, RCT	YES	NO
Gerritsen et al.,^25^ 2001	Q1	YES	NETHERLANDS	Surgical treatment of CTS	3	1,264	5,021	RCT	NO	NO
Gerritsen et al.,^26^ 2002	Q1	YES	NETHERLANDS	Non-surgical treatment carpal tunnel syndrome	4	639	3,189	RCT	NO	NO
Goodyear-Smith et al.,^27^ 2004	Q1	YES	NEW ZEALAND	Non-surgical treatment carpal tunnel syndrome	1		2,536	RCT	NO	NO
Hamamoto filho et al.,^28^ 2009	Q3	YES	BRAZIL	Anti-inflammatory drugs in the treatment of CTS	1	323	3,078	RCT	NO	NO
Hu et al.,^29^ 2016	Q2	YES	CHINA	Open versus endoscopic release	2	142	3,052	RCT	YES	YES
Huisstede et al.,^30^ 2010	Q1	YES	NETHERLANDS	Effects of non-surgical treatment on CTS	1	4,596	5,822	SYSTEMATIC REVIEW/RCT	NO	NO
Huisstede et al.,^31^ 2010	Q1	YES	NETHERLANDS	Effects of surgical treatment on CTS	1	2,957	9,127	SYSTEMATIC REVIEW/RCT	NO	NO
Huisstede et al.,^32^ 2017	Q1	YES	NETHERLANDS	Effectiveness of physiotherapy and electrophysical modalities in CTS	2	1,617	5,273	REVIEWS, RCT	NO	NO
Huisstede et al.,^33^ 2017	Q1	YES	NETHERLANDS	Comparison between different treatment modalities and post-surgical interventions	2	9,566	7,352	REVIEWS, RCT	NO	NO
Huisstede et al.,^34^ 2018	Q1	NO	NETHERLANDS	Oral pain medications versus placebo/oral steroids versus splinting/corticosteroid versus placebo	2	1,760	5,171	SYSTEMATIC REVIEW/RCT	NO	NO
Hunt et al.,^35^ 2009	Q3	YES	UNITED KINGDOM	Chiropractic manipulation CTS	1	91	2,513	RCT	NO	NO
JImenez Del Barrio et al.,^36^ 2016	Q2	YES	SPAIN	Effectiveness of non-surgical carpal tunnel syndrome treatment	2	1,818	2,505	“CLINICAL TRIALS”	NO	SIM
Kim et al.,^37^ 2019	Q2	YES	SOUTH KOREA	Shockwave therapy versus non-therapy - CTS	1	281	2,341	RCT	YES	YES
Kim et al.,^38^ 2015	Q2	NO	SOUTH KOREA	Effectiveness of nerve and tendon slip exercises in CTS	1	261	1,697	RCT	NO	YES
Klokkari et al.,^39^ 2018	Q3	YES	GREECE/CYPRUS	Surgical versus non-surgical treatment	2	1,787	5,194	RCT, CT, PROSPECTIVE, RETROSPECTIVE	YES	YES
Kohanzadeh et al.,^40^ 2012	Q2	YES	UNITED STATES	Open versus endoscopic release	3	4,178	1,846	RCT, RETROSPECTIVE	NO	NO
Lai et al.,^41^ 2019	Q1	YES	CHINA	Surgical treatment with reconstruction versus without retinaculum flexor reconstruction	1	639	2,644	RCT	YES	YES
Li et al.,^42^ 2019	Q2	YES	CHINA	Standard incision versus limited incision	2	1,020	2,722	RCT	YES	YES
Li et al,^43^ 2016	Q2	YES	CHINA/UNITED STATES	Low frequency laser effectiveness in CTS	4	531	2,112	RCT	YES	NO
Lim et al.,^44^ 2017	Q1	YES	AUSTRALIA/SINGAPORE	Mobilization of the median nerve in CTS	2	404	2,676	RCT	NO	YES
Malahias,^45^ 2019	Q2	YES	GREECE/CYPRUS	Platelet rich plasma versus control	2	192	2,636	RCT, CASE CONTROL, PROSPECTIVE CONTROLED TRIAL, CASE CONTROL, CASE REPORT	NO	YES
Marshall et al.,^46^ 2007	Q1	YES	CANADA	Corticosteroid injection into the carpal tunnel	1	671	7,052	RCT/QUASI-RCT	YES	NO
Medina McKeon et al.,^47^ 2008	Q2	NO	UNITED STATES	CTS nerve slip	1	428	4,284	CT	YES	NO
Muller et al.,^48^ 2004	Q1	NO	CANADA	Effects of non-surgical treatment on CTS	2	1,280	5,387	RCT UNTILAUTHOR’SOPINION	NO	NO
O’Connor et al.,^49^ 2003	Q1	YES	CANADA/AUSTRALIA	Non-surgical treatment in CTS (except steroid injection)	3	884	10,131	RCT/QUASI-RCT	YES	NO
O’Connor et al.,^50^ 2012	Q1	YES	AUSTRALIA/CANADA	Effects of ergonomic positioning or equipment versus no treatment, placebo, non-surgical treatment	3	105	4,654	RCT/QUASI-RCT	YES	NO
Page et al.,^51^ 2012	Q1	YES	AUSTRALIA	Orthosis versus no treatment, placebo, other non-surgical intervention	2	1,190	14,163	RCT/QUASI-RCT	YES	NO
Page et al.J,^52^ 2012	Q1	YES	AUSTRALIA	Exercise and mobilization versus placebo, no treatment or non-surgical treatment	2	741	20,024	RCT/QUASI-RCT	NO	NO
Page et al.,^53^ 2013	Q1	YES	AUSTRALIA	Therapeutic ultrasound versus other treatments for CTS	3	443	14,759	RCT	YES	NO
Piazzini et al.,^54^ 2007	Q1	NO	ITALY	Non-surgical treatment in CTS	1	1,556	2,569	RCT	NO	NO
Sanati ka et al.,^55^ 2011	Q1	YES	SCOTLAND/ UNITED KINGDOM/UNITED STATES/IRAN	Standard incision versus limited incision	6	1,512	1,697	RCT	YES	NO
Sayegh et al.,^56^ 2014	Q1	YES	UNITED STATES	Open versus endoscopic release	1	1,859	3,505	RCT	YES	YES
Scholten et al.,^57^ 2007	Q1	YES	NETHERLANDS	Different surgical treatments	1	1,284	4,137	RCT	YES	NO
Shi et al.^58^ 2011	Q2	YES	CANADA	Surgical treatment versus non-surgical treatment	1	712	2,948	RCT, CT	YES	NO
Shi et al.,^59^ 2018	Q2	YES	CANADA	Surgical intervention versus no surgical intervention	4	1,028	2,800	RCT	YES	YES
Sim et al.,^60^ 2011	Q2	YES	SOUTH KOREA/UNITED KINGDOM	Acupuncture versus other non-surgical treatments	4	442	2,245	RCT	YES	NO
Soltani et al.,^61^ 2013	Q1	YES	UNITED STATES	CTS recurrence: open decompression versus flap	1	658	2,990	CASE SERIES: PROSPECTIVE/RETROSPECTIVE	YES	NO
Thoma et al.,^62^ 2004	Q1	NO	CANADA	Open versus endoscopic release	1		2,448	RCT	NO	NO
Vasiliadis et al.,^63^ 2014	Q1	YES	GREECE/SWEDEN/CANADA/NETHERLANDS	Endoscopic release versus other surgical intervention in CTS	4	2,586	11,843	RCT/QUASI-RCT	YES	NO
Vasiliadis et al.^64^ 2015	Q1	YES	SWITZERLAND/GREECE/CANADA/UNITED KINGDOM/NETHERLANDS	Open versus endoscopic release	6	2,449	4,754	RCT/QUASI-RCT	YES	YES
Verdugo et al.,^2^ 2008	Q1	YES	CHILE	Surgical treatment versus non-surgical treatment	1	198	3,276	RCT/QUASI-RCT	YES	NO
Wade et al.,^65^ 2018	Q1	YES	UNITED KINGDOM/ITALY	Absorbable versus non-absorbable suture	4	255	9,703	RCT/QUASI-RCT	YES	YES
Zuo et al.,^66^ 2015	Q2	YES	CHINA	Open versus endoscopic release	1	1,253	3,940	RCT	YES	NO

PRISMA = preferred reporting items for systematic reviews and meta-analyzes; CTS = carpal tunnel syndrome; RCT = randomized controlled trial; CT = controlled trial.

**Table 2.** Quantitative data

	Average	CI 95%
PRISMA E1	12.67	11.36–13.99
PRISMA E2	14.00	12.94–15.06
PRISMA average	13.34	12.17–14.5
AMSTAR E1	7.17	6.38–7.96
AMSTAR E2	7.21	6.59–7.83
AMSTAR average	7.19	6.51–7.87
Number patients	1326.66	905.74–1747.58
Number words	4872.27	3.867.37–5877.18

CI = confidence interval; E1 = examiner 1; E2 = examiner 2.

The mean values of the two examiners (Examiner E1 and Examiner E2) for the PRISMA and AMSTAR scores were compared with the following covariates: impact factor, conflict of interest, country of origin, meta-analysis, cite PRISMA, and design of the included studies.

Considering the stratification within the AMSTAR, 87% of the studies evaluated by E1 had “low” or “very low” quality, whereas for E2, this value was 76.4%. Thus, only 2.7% of the studies were classified as having “high” quality (Table 3).

**Table 3.** Qualitative results of the A measurement tool to assess systematic reviews assessment

	E1		E2		Average E1; E2	
“Very low” quality	26	(47%)	17	(31%)	21.5	(39.1%)
“Low” quality	22	(40%)	25	(45.4%)	23.5	(42.7%)
“Moderate” quality	5	(10%)	12	(21.8%)	8.5	(15.5%)
“High” quality	2	(3%)	1	(1.8%)	1.5	(2.7%)

E1 = Examiner 1; E2 = Examiner 2.

PRISMA resulted in the highest scores when the studies included meta-analysis (15.61 versus 10.40; P = 0.008). There were no differences in the other variables analyzed, as shown in Table 4.

**Table 4.** Comparison of covariates for PRISMA

	Number of studies (total = 55)	Average	CI	P value
Impact factor	Q1 (n = 34)	14.16	12.64–15.68	0.095
Meta-analysis	Yes (n = 31)	15.61	14.25–16.97	0.008
Study design	RCT (n = 33)	14.47	12.82–16.12	0.103
Conflict of interest	Conflict (n = 46)	13.95	12.69–15.21	0.155
Country of origin	China (n = 6)	14.67	12.96–16.38	0.268
Quote PRISMA	Yes (n = 17)	14.47	12.19–16.75	0.131

CI = confidence interval; PRISMA = preferred reporting items for systematic reviews and meta-analyzes; RCT = randomized controlled trial.

AMSTAR resulted in higher scores when the studies performed meta-analysis (8.43 versus 5.59; P = 0.009) or when they included randomized clinical trials (RCT) (7.95 versus 6.06; P = 0.043), as presented in Table 5.

**Table 5.** Comparison of Covariates for A Measurement Tool to Assess Systematic Reviews

	Number of studies (total = 55)	Average	CI	P value
Impact factor	Q1 (n = 34)	7.55	6.68–8.42	0.372
Meta-analysis	Yes (n = 31)	8.43	7.55–9.31	0.009
Study design	RCT (n = 33)	7.95	6.98–8.92	0.043
Conflict of interest	Conflict (n = 46)	7.57	6.83–8.31	0.173
Country of origin	China (n = 6)	8.21	6.72–9.7	0.16
Quote PRISMA	Yes (n = 17)	7.47	6.04–8.9	0.183

CI = confidence interval; PRISMA = Preferred Reporting Items for Systematic Reviews and Meta-Analyses; RCT = randomized controlled trial.

Journals with the greatest impact did not influence most variables, except for the PRISMA citation statement. In publications that cited PRISMA, 47.6% were low-impact journals and 20.6% were high-impact journals. Among those that did not mention PRISMA, 52.4% were low-impact journals, whereas 79.4% were high-impact journals (P = 0.035), as shown in Table 6.

**Table 6.** Impact factor X covariates

		Low impact(Non Q1)		High impact(Q1)		Total		P value
		n*	%	n*	%	n*	%	
Quote PRISMA	Yes	10	47.60%	7	20.60%	17	30.90%	0.035
Conflict of interest	Conflict	18	85.70%	28	82.40%	46	83.60%	0.743
Study design	RCT	18	85.70%	31	91.20%	49	89.10%	0.528
N. Institutions	Multicentric	13	61.90%	20	58.80%	33	60.00%	0.821
Meta-analysis	Yes	12	57.10%	19	55.90%	31	56.40%	0.927
Country of origin	China	4	19.00%	2	5.90%	6	10.90%	0.128

*Total number of studies = 55. PRISMA = Preferred Reporting Items for Systematic Reviews and Meta-Analyses; RCT = randomized controlled trial.

By assessing the correlation of the country of origin with the same qualitative covariates, we observed a positive correlation between Chinese studies and those that performed meta-analysis (100% in Chinese studies versus 51% in non-Chinese studies) (P = 0.022), as presented in Table 7.

**Table 7.** Country of origin X covariates

		Chinese studies		Chinese studies		Total		P value
		n*	%	n*	%	n*	%	
Quote PRISMA	Yes	3	50%	14	28.60%	17	30.90%	0.284
Conflict of interest	Conflict	5	83.30%	41	83.70%	46	83.60%	0.983
Study design	RCT	6	100%	43	87.80%	49	89.10%	0.364
N. Institutions	Multicentric	3	50%	30	61.20%	33	60.00%	0.596
Meta-analysis	Yes	6	100%	25	51.00%	31	56.40%	0.022

*Total number of studies = 55. PRISMA = Preferred Reporting Items for Systematic Reviews and Meta-Analyses; RCT = randomized controlled trial.

We identified that the intraobserver correlation for E1 and E2 in the AMSTAR and PRISMA scores was above 0.8, with perfect agreement between the pairs, as presented in Table 8.

**Table 8.** Intra-observer correlation between the scores for A Measurement Tool to Assess Systematic Reviews and Preferred Reporting Items for Systematic Reviews and Meta-Analyzes

		E1	E2	Average
Spearman	Corr (r)	0.82	0.798	0.829
	P value	< 0.001	< 0.001	< 0.001
ICC	Corr (r)	0.856	0.839	0.857
	P value	< 0.001	< 0.001	< 0.001

ICC = intraclass correlation coefficient; E1 = examiner 1; E2 = examiner 2.

The inter-observer correlation between the two examiners, using the Blant–Altman model, showed that PRISMA has low reliability, unlike AMSTAR, as the values of the latter were closer to zero, as shown in Table 9.

**Table 9.** Inter-observer correlation between the scores for a PRISMA and AMSTAR

	PRISMA	AMSTAR
Average	-1.33	-0.04
Standard deviation	1.91	1.48
P value	< 0.001	0.856
Regression	< 0.001	0.001

PRISMA = Preferred Reporting Items for Systematic Reviews and Meta-Analyses; AMSTAR = A Measurement Tool to Assess Systematic Reviews.

Applying the Kappa coefficient to assess inter-observer agreement in AMSTAR, revealed substantial agreement (0.61–0.8) when grouping this tool into two variables: “low” or “medium/high” quality studies, as presented in Table 10.

**Table 10.** Inter-observer correlation for A Measurement Tool to Assess Systematic Reviews

	Kappa	P value
Original*	0.442	< 0.001
Grouped^**^	0.641	< 0.001

*Very low, low, moderate, high; **Low, moderate/high.

Multivariate analysis using the linear regression model showed a greater impact factor for a journal when a study used meta-analysis, and further showed that multicenter studies have significantly increased PRISMA and AMSTAR scores, as presented in Table 11.

**Table 11.** Results of multivariate analysis

	PRISMA		AMSTAR	
	Coef. (B)	P value	Coef. (B)	P value
Constant	4.89	0.03	3.36	0.016
Impact Q1	2.54	0.01	1.06	0.076
Meta-analysis	4.74	< 0.001	2.53	< 0.001
Multicentric	2.2	0.022	1.69	0.005
Conflict of interest	1.41	0.263	1.16	0.138
Non-Chinese studies	0.17	0.911	-0.45	0.633
Quote PRISMA	1.12	0.275	-0.2	0.747
RCT	1.34	0.353	0.26	0.77
ANOVA	< 0.001		< 0.001	
R2	54.40%		49.20%	

PRISMA = Preferred Reporting Items for Systematic Reviews and Meta-Analyses; AMSTAR = A measurement tool to assess systematic reviews; Coef. = coefficient; RCT = randomized controlled trial; ANOVA = analysis of variance.

## DISCUSSION

Systematic reviews on CTS are mostly of low quality. Several factors are related to better methodological quality, including study design, studies that mention PRISMA, and meta-analyses. Factors such as conflicts of interest, country of origin, and multicenter studies did not have the same influence.

Similar studies have shown consistent results regarding the intra-observer correlation of the PRISMA and AMSTAR scores. In agreement with our study, these studies found the influence of the presence of meta-analysis on the score values. They also pointed out that there was no difference in the AMSTAR score in terms of the presence of conflicts of interest and impact factor.^11^

Other studies have indicated that reviews including only RCTs have better AMSTAR scores, which is similar to the findings of our study. They also observed differences in the PRISMA results of studies that presented declared conflicts of interest. In our study, we did not observe this difference.^67^

There have been relatively few studies on the quality of systematic reviews of specific hand and upper limb diseases in orthopedics. However, several of these studies have pointed out that the quality of systematic reviews in leading journals in orthopedics is suboptimal,^68-70^ despite having substantially improved following publication of PRISMA.^71^

Taking into account the same area of knowledge of hand surgery, an overview of the quality of systematic reviews of the treatment of fractures of the distal radius^9^ also showed that studies only including randomized clinical trials and those that performed meta-analyses had better quality.

AMSTAR scores had greater inter-observer agreement than PRISMA scores, especially when dichotomously dividing the qualitative results into high- and low-quality studies. Our findings therefore suggest that AMSTAR is more robust, although improvements are still possible.

PRISMA has emerged as a guideline for systematic reviews with better technical quality, which differs from the AMSTAR scores. We speculate that this is one explanation for the lower agreement between observers and the lower robustness of this score. In addition, AMSTAR generally presents more detailed items.^7-9,67^

Observing the relationship between the same covariates and country of origin, we noted that Chinese studies performed meta-analyses more consistently: 100% of Chinese studies included in this study performed meta-analyses, while only 51% of non-Chinese studies performed meta-analyses in their systematic reviews, which supports the current trend of high-quality Chinese studies.^11^

Studies citing PRISMA were more common in journals with a lower impact factor. Although this finding is not intuitive, many high-impact journals endorse PRISMA, and we inferred that many high-quality studies rely on the items in this questionnaire despite not explicitly quoting it (i.e. they have a high PRISMA score despite not mentioning it).

Systematic reviews on CTS have consistently revealed recurrent imperfections. Many lost points on PRISMA for presenting an incomplete, unstructured summary, not presenting a review protocol, not presenting a detailed search strategy, not presenting the data combination methods in detail, and not presenting the impact of the risk of bias on the results. Studies lose points in the AMSTAR score for not explaining the study designs included, not describing the studies in detail, not citing the study funding, not discussing the impact of the risk of bias of the studies on the results, and not explaining the causes of heterogeneity between studies. An ideal systematic review of CTS would explain all of these aspects.

The use of PRISMA and AMSTAR is important for the generation of quality scientific evidence, and allows for the critical evaluation of available publications to date. The dissemination of other similar systems allows for the organization and systematization of the main aspects related to the quality and reliability of information sources. This would further improve the refinement of the best currently available evidence for the treatment of carpal tunnel syndrome.

### Limitations

The main limitation of this study was that the search for systematic reviews was published in all languages, except Mandarin.

We tried to minimize biases in the selection, application of questionnaires, and data analysis by carrying out our analysis with independent examiners, and any disagreements were concluded with reference to the senior author. Statistical analysis was conducted by an independent statistician with no conflicts of interest.

## CONCLUSIONS

Our results suggest that published systematic reviews on the treatment of CTS are of low quality, and those that contain meta-analyses and include randomized clinical trials are generally of better quality.

The PRISMA and AMSTAR scores are effective tools for formulating and guiding systematic reviews, although AMSTAR performed better. The reproducibility of AMSTAR scores allows for the analysis of future studies on the treatment of CTS, which is useful for the preparation of other high-quality studies.
